# The Association Between Fitness Test Scores and Musculoskeletal Injury in Police Officers

**DOI:** 10.3390/ijerph16234667

**Published:** 2019-11-23

**Authors:** Liana Lentz, Jason R. Randall, Christine A. Guptill, Douglas P. Gross, Ambikaipakan Senthilselvan, Donald Voaklander

**Affiliations:** 1 School of Public Health, University of Alberta, Edmonton, AB T6G 1C9 Canada; jason.randall@ualberta.ca (J.R.R.); sentil@ualberta.ca (A.S.); dcv1@ualberta.ca (D.V.); 2 Faculty of Rehabilitation Medicine, Department of Occupational Therapy, University of Alberta, Edmonton, AB T6G 2G4, Canada; guptill@ualberta.ca; 3 Faculty of Rehabilitation Medicine, Department of Physical Therapy, University of Alberta, Edmonton, AB T6G 2G4, Canada; dgross@ualberta.ca

**Keywords:** work, physical fitness, exercise test, risk factors, musculoskeletal diseases

## Abstract

A police officer’s career is hazardous and physically demanding. In order to perform occupational tasks effectively and without injury, officers require adequate physical abilities. The aim of this study was to investigate the relationship between scores on several fitness tests and musculoskeletal injury in a group of municipal police officers. This retrospective study used existing data to examine the relationship between risk of injury and fitness test performance. Injured and uninjured police officers scored significantly differently on several fitness measures. A multivariate regression indicated that a combination of age, sex, number of pull ups completed and maximal oxygen consumption (VO_2max_) best explained injury risk. Additionally, the findings indicated an interaction between sex and VO_2max_, and so the effect of VO_2max_ on injury risk cannot be understood without accounting for sex.

## 1. Introduction

Police officers perform a wide range of tasks while upholding their duty to protect the public and prevent crime. Even though many of a police officer’s daily duties are sedentary, this career is hazardous and physically demanding. At any given time, there is potential that a police officer will be involved in a critical incident fighting for their life or protecting the life of another. In order to perform occupational tasks effectively and without injury, officers require adequate physical abilities and readiness.

Physical fitness describes a concept that is different from, and a component of, occupational physical ability or physical readiness. It has been defined in many ways. A general definition relates to the ability to carry out daily tasks without fatigue, leaving energy for leisure pursuits and to meet the physical stresses required in an emergent situation [1]. Physical fitness is a set of attributes that is related to health, which can be measured with specific tests and can be broken down into the components of cardiorespiratory endurance, muscular endurance, muscular strength, body composition, and flexibility [2]. Police officers are expected to have an above average level of physical fitness to ensure that they can fulfill their duty to protect the community when involved in a critical incident. If officers are unable to perform their duties or are away from work because of injury or illness, the community is affected by decreased service, increased response time to emergencies, and increased cost of maintaining police services.

The economic and personnel costs of injuries has been an impetus for initiating employee fitness and wellness programs in a variety of organizations and evidence suggests that such programs may decrease injuries, absenteeism, and the cost of group health care benefits [3,4]. Police officers who participate in fitness activities appear less likely to have an injury [5,6]. Physical activity has also been found to protect against the negative effects of psychological stress [7], improve sleep [8] and may also lend to decreases in depression [9]. There has also been a significant association reported between lower aerobic fitness and increased injury risk [10,11,12]. Being responsible for public safety, police officers have a psychologically and physiologically demanding job and must have good mental and physical health in order to do their duty.

Police officer injury is an important public health problem not only because the incidence of injuries to police officers is higher than in most other occupations [13] but also because having police officers absent from work affects the safety of the community. On average, in the U.S. and Australia, police have been found to have an injury rate three times greater than that for all other workers [14,15]. In one municipality, police injury accounted for 48% of all emergency responder injuries compared to fire and emergency medical services who reported 36% and 18% of the total injuries respectively [16].

Research examining the relationship between physical fitness test scores and the occurrence of musculoskeletal injuries (MSIs) in police officers is very inadequate. In a recent systematic review, only four studies examining the relationship between fitness and injury in police officers were identified [17]. Therefore, evidence of relationships between many aspects of physical fitness and occupational injury is limited.

The aim of this study was to investigate the relationship between scores on several fitness tests and musculoskeletal injury in a group of municipal police officers. The main hypothesis was that the mean scores on fitness tests would differ between injured and uninjured police officers. We investigated the risk of injury and fitness test performance to identify whether different components of fitness have differing relationships to injury.

## 2. Materials and Methods

### 2.1. Research Design

This was a retrospective study of active police officers in a municipal police service in Western Canada. The study received ethical approval from the University of Alberta Ethics Review Board. The yearly mean number of officers in this service during the study period was 1674 [18]. A secondary data analysis was completed to examine the relationship between physical fitness and injury in active police officers. Fitness data included fitness test scores from annual fitness testing that were recorded at the time of testing in Excel by fitness unit employees (see Appendix A for more details of the fitness test). Fitness unit employees were employed by the police service and were either certified through the Canadian Society of Exercise Physiologists or the National Strength and Conditioning Association. Any MSIs that affected a police officer’s ability to work in full capacity, including those injuries that were not work-related, were self-reported to the police service. Injury data was recorded by the police service using specialized injury report software. Data were extracted from these databases by the data manager. To maintain anonymity, an employee of the police service removed names and changed identification numbers in the data sets prior to providing the data to the researchers. The data were obtained with permission from the police service and according to Alberta’s Freedom of Information and Protection of Privacy Act. 

### 2.2. Sampling

The subjects included all active police officers in the police service who completed annual fitness testing between 1 January 2013 and 2 June 2016. Since fitness testing is mandatory until the age of 45, only those police officers that were 45 years of age and under during the observation period were included. During this time period, officers complete more than one fitness test. However, the scores for only one fitness test per subject was included in the analysis. For injured officers, scores from the last fitness test prior to injury were used. In total, data on 1006 subjects were available for the study.

### 2.3. Fitness Variables

The annual fitness test consisted of several field tests and included measures of body mass, body fat percentage, hand grip strength, vertical jump height, leg power, pull-ups, kilograms pulled, push-ups, plank time, and maximal oxygen consumption (VO_2max_). VO_2max_ was measured in 1 of 2 different ways during testing, either indirectly measured using the 20 m shuttle run test or directly measured using a cycle or treadmill ergometer. Less than 10% of subjects had VO_2max_ directly measured. Research indicates that the results of either measure are comparable and valid measures of VO_2max_ [19,20]. The mean scores between the shuttle run and direct measure did not vary significantly, and so the values were combined in the analysis. Since 90% of participants were tested using the same method of estimating VO_2max_ and the correlation between various methods of VO_2max_ is very high, comparisons between individuals should be valid. The 20 m shuttle run qualifies legally as a bona fide occupational test for physically demanding public safety occupations and therefore is considered an appropriate and accurate measure for this study [21].

### 2.4. Definition of Injury

For the purpose of this study, musculoskeletal injury is defined as any injury to a joint or bone identified in the medical data with diagnoses of dislocation, fracture, knee ligament injury, knee meniscus tear, non-specific pain, or sprain/strain. Any duplicate data were identified based on the participant number, injury date, and body part injured and were removed prior to analysis. Only the first recorded injury was included in the analysis.

### 2.5. Analysis of Data 

A descriptive analysis included means and standard deviations for continuous variables (fitness scores) and proportions and frequencies for discrete variables (sex, injury status, injury diagnosis, injury site). The significance of the differences in means was examined using a student’s t-test. Odds ratios (OR) and 95% confidence intervals (CI) were used to compare risks between groups. A *p*-value of 0.05 or less was considered significant.

A matched case-control analysis was conducted to determine the relationship between fitness test scores and musculoskeletal injury (MSI). Participants had a staggered entry in to the study and fitness test date was a time dependent variable in relation to injury. To adjust for possible temporal variation in training protocols or work activity, each case was matched on the date of fitness test, with 2 controls. For cases, the fitness test used in the analysis was the test completed just prior to injury. Controls were matched with cases by a fitness test within ±30 days of the date that fitness testing was complete by their matched cases. Injury was a dichotomous independent variable (injured or not injured) when multivariable logistic regression was used to determine the effects of fitness scores on injury. Initially, univariate OR and 95% CI were estimated for each of the fitness measures. For the multivariable analysis, purposeful selection method was used for the model building [22]. Effect modification was explored between the variables in the main effects model and any significant interaction terms were included in the final model. The sample size available was adequate to determine an odds ratio of approximately 1.20 or greater for the relationship between fitness test scores and musculoskeletal injury with a level of significance 0.05 and 80% statistical power. All statistical analyses were performed on SAS^®^ software, Version 9.4 for Windows (SAS Institute Inc., Cary, NC, USA).

## 3. Results

### 3.1. Descriptive Analysis

**Figure 1:**
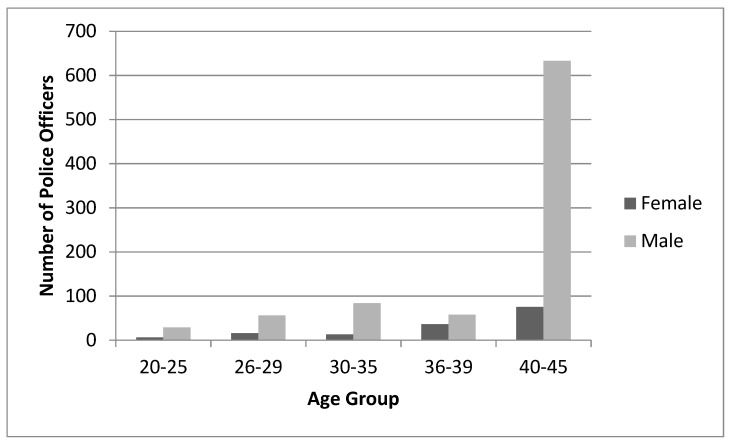
Distribution by age group and sex.

Initially, there were 1357 subjects and 481 injuries. After matching, 1006 subjects including 336 injured subjects remained for analysis. Subjects had a mean age of 39.7 years (SD = 5.8), where the greatest proportion of the police officers were over 40 years of age (Figure 1). Females accounted for 14.5% of the subjects and had a mean age of 38.4 (SD = 6.3) years, whereas males had a mean age of 40.0 (SD = 5.7) years. Years of service was similar between sexes, where females had an average of 14.4 (SD = 9.0) and males had an average of 14.1 (SD = 6.6) years of service. During the study period, there were 336 new MSIs reported and 89.3% of these injuries were diagnosed as sprains and strains. Females accounted for 17.9% of the injured subjects.

### 3.2. Difference in Mean Scores

The mean fitness test scores for all subjects are summarized in Table 1. Not all the subjects did all the fitness tests, and so sample sizes (*n*) are shown to describe the missing data. There were significant differences in fitness test scores between injured and uninjured subjects in all measures except body fat percentage, body mass, heart rate, left grip strength, and combined grip strength. Injured subjects scored higher on fitness tests including right hand grip strength, vertical jump, leg power, pull ups, kilograms pulled, pushups, speed, maximal oxygen consumption (VO_2max_), and plank time.

### 3.3. Logistic Regression Analysis

The univariate analysis indicated that age, sex, vertical jump height, leg power, number of pull ups completed, kilograms pulled, number of push-ups completed and VO_2max_ were significantly associated with experiencing MSI (Table 2). The multivariable logistic regression analysis indicated that the main effects model that was significant and best described increased injury risk included decreased age, female sex, decreased number of pull ups, and increased VO_2max_ (Table 3). The predictor variables in the final model were centered around the mean. The measure of the effect of VO_2max_ on injury was significantly modified by sex, and so the interaction between sex and VO_2max_ was also included in the final model. For a one-unit increase in VO_2max_, females were 1.59 times more likely to have an injury, whereas males were 0.97 times less likely to have an injury.

## 4. Discussion

### 4.1. Findings

The objective of this study was to determine the relationship between several fitness test scores and the risk of musculoskeletal injury in a group of municipal police officers in Western Canada. A multivariate regression indicated that a combination of decreased age, female sex, decreased number of pullups, and increased VO_2max_ best explained increased injury risk. The number of pull-ups and VO_2max_ are general indicators of strength and aerobic fitness that may be related to a variety of MSIs, although specific injury mechanisms are not clear at this point due to limitations in available data. Additionally, the findings indicated an interaction between sex and VO_2max_, and so the effect of VO_2max_ on injury risk cannot be understood without accounting for sex.

### 4.2. Fitness and Injury

Some police studies have found that officers who engage in fitness training were less likely to experience an injury reportable to occupational health [23] and police officers with the highest self-reported fitness levels were less likely to experience sprains or strains than those who considered themselves less fit [6]. In contrast, it has also been reported that police officers who collected workers’ compensation were more fit than those who did not [24]. The conflicting findings could be due to several factors including differing methodologies and varying definitions of fitness level, physical activity level, and/or injury. The current study found that an increased fitness level and increased VO_2max_ was a risk factor for injury within females. However, this finding should be further investigated. Research investigating the mechanism of injury in police, military, and firefighters has highlighted that participation in sport or fitness activities is a common mechanism for injury in these groups and account for approximately 30% of non-work-related injuries [25,26,27,28]. Other injury research involving healthy adults indicates that participating in physical activity, including higher volumes of aerobic exercise (300 min/week-1), is associated with an increased risk of both acute MSIs and recurrent MSIs [29] and that the risk of sustaining an activity-related injury is increased with a higher duration of physical activity per week [30]. The proportion of healthy men and women with activity-related injury also increased with higher cardiovascular fitness levels and highly fit men had almost four times the risk of MSIs compared to that of men in the lowest cardiorespiratory fitness category [30].

Nabeel et al. (2007) classified people with a high level of physical activity as exercising at least 30 min twice per week [6] and found that police officers who were more physically active were more resistant to injury. However, this definition of activity does not even meet the “low-dose aerobic exercise” definition used by Brown et al. (2017) who found a dose-response relationship between physical activity and injury risk [29]. Given that increased physical activity (in duration and intensity) increases cardiorespiratory fitness (i.e., VO_2max_), this may explain the conflicting information regarding the relationship between physical activity and injury as well as the association between increased VO_2max_ and injury risk observed in this study. It may be exposure to the exercise that increased injury risk, rather than injury being a result of high cardiovascular fitness.

### 4.3. Pull Ups

Very little research has investigated the relationship between pull ups and occupational injury. Two studies involving male military trainees [31,32] and one examining Federal Bureau of Investigation trainees [26] concluded that there was no association between the number of pull ups performed and risk of MSIs. Another study indicated a significant univariate association between increased pull ups completed and a decreased risk of MSIs in male and female British army recruits [33]. In a more recent study, Swedish Armed Forces marines entering the training course who performed fewer than four pull-ups were at increased risk for lower back pain (HR 1.9, 95% CI 1.2 to 3.0) [34]. No studies were located investigating this relationship in active duty police officers or police recruits. In the current study, an increased number of completed pull ups was indicated in the main effects model as being protective of injury (OR 0.89, 95% CI 0.80 to 0.98). However, this significance disappeared in the interaction model.

### 4.4. Age

Increased age has been found to be a risk factor for injury in the general working population [35,36,37]. However, for police officers, this does not appear to be reflected as increased age appears to be a protective factor for injury [15,38,39]. This was also indicated in the current study where increased age was associated with a decreased injury risk (OR 0.55; 95% CI 0.50–0.61). This decreased risk with increased age may be a proxy for the task differences between junior and senior police officers.

Police officers’ work-related injuries primarily occur when apprehending and detaining a noncompliant or assaultive suspect [40] and can account for 31.5% to 61.7% of officer injuries [27,41]. The likelihood of apprehending suspects is related to the position in which an officer works. Officers working in front line positions (i.e., patrol) are more likely to apprehend suspects than those working in more investigative or administrative areas. In the participating police agency, patrol is the first position that new officers work in once they finish recruit training and often remain there for a minimum of 3 to 5 years. Research supports that it is during these first five years that police officers are most likely to get injured at work [23]. This is likely why younger police officers and those with fewer years of service are more likely to become injured, because their daily duties put them at increased risk for injury through greater exposure to suspects and the opportunity to arrest them. The number of years served was not included in the analysis because it was highly correlated with age and had a strong relationship with injury. The main research question was examining the relationship between fitness and injury, but years of service cannot be entirely ignored. It has also been suggested that the risk of injury associated with age is different between sexes. In a previous study examining the relationship between sex, age and injury, younger females (age 20–29) had more time loss claims but this difference was modified for older police officers. At age 30, as age increased, males continued to have a greater proportion of injuries compared to females [42]. The current study did not identify a significant interaction between age and sex in relation to injury (*p* = 0.15).

### 4.5. Sex

Overall, the relationship between sex and injury potential in police has not been well investigated. This may be due to females being a minority in this occupation. For example, in 2017, only 12.5% of law enforcement officers in the United States were female [43], whereas, in Canada, females accounted for 21% of all sworn officers during this same time [18]. As more women enter policing, the number of female police officers who are hurt on the job appears to increase [42]. However, as the number of female police officers is generally low, small changes can result in large percentage changes falsely indicating that females are more likely to become injured than males. Proper comparisons and careful evaluation of data need to be made. It is possible that the increased likelihood of injury for females may not be related to their roles or performance as police officers but to other activities external to their job such as sport or fitness activities [25,44].

### 4.6. Limitations

A limitation of this study, which is common to studies using secondary data, is that the information was recorded for a reason other than research. Additionally, there was no information regarding the mechanism of injury. Though the injuries recorded affected the police officer’s ability to work at full capacity, information regarding how the officer became injured was not available.

The definition of injury can influence the apparent relationship between fitness measures and injury in police officers. In this study, and the literature referred to in this paper, we have referred to MSIs. MSIs involve damage to bone, ligaments, tendons, muscle and cartilage. In research, injures are identified through self-report [6,25], workers’ compensation claims [23], or reports to the employer [28,45]. Injuries that occur during physical activity may or may not be reported to workers’ compensation but may be reported to the employer if the injured worker is not able to work to their full capacity. This lack of a common definition limits the ability to directly compare study results.

## 5. Conclusions

The objective of this study was to determine the relationship between several fitness test scores and risk of musculoskeletal injury in a group of municipal police officers in Western Canada. A multivariate regression indicated that a combination of age, sex, number of pull ups completed and VO_2max_ best explained injury risk. Additionally, the findings indicated an interaction between sex and VO_2max_, and so the effect of VO_2max_ on injury risk cannot be understood without accounting for sex.

Including the analysis of injury mechanism would also clarify the relationship between fitness and work-related injury. The fitness tests included in this study were general fitness tests. Examining the relationship between occupational-specific fitness test performances in relation to work-related injury is also recommended. Looking in more detail at the relationships between injury mechanism, fitness and occupational-specific fitness test performances will add insight into prevention strategies.

## Figures and Tables

**Table 1:** Distribution of fitness test scores by uninjured and injured.

		Uninjured					Injured				
Test	*n*	Mean	SD	Minimum	Maximum	*n*	Mean	SD	Minimum	Maximum	*p*-Value
Body Mass (kg)	661	87.20	12.05	60.53	128.40	332	86.34	13.93	52.72	165.30	0.34
Body Fat (%)	670	20.50	6.17	10.30	44.70	332	20.24	6.95	4.40	43.00	0.56
Heart Rate (bpm)	667	74.81	10.57	48.00	95.00	323	74.88	13.25	47.00	118.00	0.94
Grip Strength Right (kg)	670	48.12	10.25	32.00	78.00	336	51.75	12.31	18.00	86.00	0.0001
Grip Strength Left (kg)	670	50.85	10.86	28.00	75.00	335	49.56	12.19	12.00	84.00	0.10
Grip Strength (kg)	670	98.97	20.54	60.00	153.00	335	101.39	24.00	30.00	169.00	0.12
Vertical Jump (inches)	667	108.92	5.72	93.50	117.00	323	110.8	6.96	90.50	128.00	<0.0001
Leg Power (Watts)	667	4820.98	847.89	2247.62	6930.40	319	5167.34	1490.50	318.98	24407.39	<0.0001
Pull Ups (*n*)	641	4.45	5.69	0.00	15.00	316	6.94	5.81	0.00	24.00	0.0001
Amount Pulled (kg)	641	384.41	490.41	0.00	1295.19	313	593.07	487.87	0.00	1915.43	0.0001
Push Ups	669	28.7	11.24	10.00	54.00	324	32.49	10.75	0.00	67.00	0.0001
Speed (km/hr)	471	11.79	1.14	10.00	13.50	300	11.98	1.01	8.25	14.50	0.02
VO_2 Max_ (mL/kg/min)	508	42.24	5.86	32.59	53.60	316	44.02	6.70	20.58	59.60	0.0001
Plank Time (min)	670	2.56	1.02	0.77	5.00	328	2.69	0.91	0.13	5.73	0.04

**Table 2:** Univariate results from logistic regression.

Variable	OR	95% CI	*p*-Value
Age (years) *	0.51	0.44–0.59	<0.0001
Sex (female)	1.48	1.03–2.12	0.034
Body Mass (kg)	1.00	0.99–1.01	0.327
Body Fat (%)	0.99	0.97–1.02	0.510
Combined Grip (kg)	1.01	1.00–1.01	0.061
Vertical Jump (inches)	1.05	1.03–1.07	<0.0001
Leg Power (Watts)	1.00	1.00–1.00	<0.0001
Pull Ups (*n*) *	1.04	1.02–1.05	<0.0001
Kg Pulled	1.00	1.00–1.00	<0.0001
Push Up (*n*)	1.04	1.02–1.05	<0.0001
Vo_2max_ (mL/kg/min) *	1.07	1.04–1.09	<0.0001
Plank Time (min)	1.15	0.99–1.32	0.636

* Mean centered.

**Table 3:** Results from the multivariable logistic regression.

Variable	Odds Ratio	95% Confidence Limits	
Age (years) *	0.55	0.50	0.61
Sex (female)	1.77	0.56	5.58
Pull Ups * (*n*)	0.99	0.96	1.02
VO_2max (_mL/kg/min) *	Male 0.97	0.92	1.02
	Female 1.59	1.32	1.91

* Mean centered.

## Appendix A

Body mass was measured and recorded in pounds and converted to kilograms as metric measures are required for calculations of maximal oxygen consumption and leg power.

Percent body fat was measured through bioelectric impedance using the *Inbody* 520 body composition analyzer. Bioelectric impedance sends a low-level electrical current though the body which travels at different speed through different body tissues. The rate at which the current passes through the body is used to calculate the body’s fat free mass and thus the body fat percentage.

Grip strength was measured bilaterally using a *Smedly* hand dynamometer 12-028IJ. The subject holds the dynamometer in the hand to be tested, with their elbow at a right angle by the side of the body. The base should rest on heel of palm and the handle should rest on middle of four fingers. When ready the subject squeezes the dynamometer with maximum effort for approximately 5 s. No other body movement is allowed. The highest measure of three tests for each hand was used and added together to get the grip strength value.

Vertical jump height was measured using a *Vertec* device which is comprised of plastic swivel vanes arranged in half-inch (1.25 cm) increments which are attached to a metal pole that can be adjusted to the test subject’s reach height. It requires the subject to use their dominant hand to displace the highest possible vane with an overhead arm swing at the peak of their jump indicating the jump height.

Leg power was calculated using the Sayers Equation which estimates peak power output (Peak Anaerobic Power output or PAPw) from the vertical jump;

PAPw (Watts) = 60.7 × jump height (cm) + 45.3 × body mass (kg) − 2055

Pull-ups were counted starting from a dead hang (arms are straight, and the body is unsupported). Hands faced forward and shoulder width apart. There is no kipping or assisting by movement of the lower body allowed and the chin must be raised above the bar for the pull-up to be counted.

Poundage pulled by the upper body was calculated by multiplying the subject’s weight by the number of pull-ups completed.

Number 90° push-ups were counted with cadence of 80 beats per minute. Subjects start with their chest on the ground and then perform push-ups to the cadence. The arms must fully extend at the end of the upward movement and elbows must bend to a 90° degree angle prior to the subject pushing up again. Push-ups are counted until the subject can no longer keep proper form within the cadence.

Plank time was considered the length of time a subject can hold a plank position. This position involves the subject having their elbows, forearms and toes on the ground while holding the rest of their body off the ground in a prone position. The body is to remain straight and once the subject can no longer hold the position; they are considered to have held the plank position to their maximum time.

VO_2max_ was measured in two different ways. Each participant has only one measure. Less than 10% of subjects had VO_2max_ directly measured. The mean scores between the shuttle run and direct measure did not vary, and so the values were combined in the analysis.

1. The 20 m shuttle run (*n* = 1044) test involves subjects running back and forth on a 20 m course at various speeds ranging from 7–14 km/h. The pace is set with pre-recorded audio signals emitted at specific frequencies and subjects must complete the 20 m run within the decreasing interval times. Subjects are instructed to complete as many stages as possible and the test is stopped when the individual is no longer able to follow the pace. The last stage completed is recorded.
  Retro-extrapolation from the 20 m shuttle run stage completed using the Leger formula;
  31.025+(3.238×shuttle run stage)−(3.248×18)+(0.1536×shuttle run stage×18)
2. Direct measure VO_2_ max (*N* = 51) was measured by putting a face mask on the subject and directly measuring the volume and gas concentrations of inspired and expired air. The test involves either exercising on a treadmill or a bike at an intensity that increases every few minutes until exhaustion and is designed to achieve a maximal effort.

When compared to direct measures of aerobic power, the 20 m shuttle run using the Leger formula is a valid and reliable method for estimating aerobic power [19,20].
